# Metabolomics-based analysis of the diatom *Cheatoceros tenuissimus* combining NMR and GC–MS techniques

**DOI:** 10.1016/j.mex.2024.102695

**Published:** 2024-04-03

**Authors:** Afrah Alothman, Abdul-Hamid Emwas, Upendra Singh, Mariusz Jaremko, Susana Agusti

**Affiliations:** a King Abdullah University of Science and Technology (KAUST), Biological and Environmental and Science and Engineering, Marine Science Program, Thuwal, 23955-6900, Saudi Arabia; b King Abdullah University of Science and Technology (KAUST), Core Labs, Thuwal, 23955-6900, Saudi Arabia; c King Abdullah University of Science and Technology (KAUST), Biological and Environmental and Science and Engineering, Thuwal, 23955-6900, Saudi Arabia

**Keywords:** Optimizing extraction procedure and combining NMR with GC–MS techniques for comprehensive metabolome analysis of microalgae, Metabolomics, NMR, GC–MS, Microalgae, Diatoms, Extraction, Metabolites profiling

## Abstract

Metabolomics, a recent addition to omics sciences, studies small molecules across plants, animals, humans, and marine organisms. Nuclear magnetic resonance (NMR) and gas chromatography-mass spectrometry (GC–MS) are widely used in those studies, including microalgae metabolomics. NMR is non-destructive and highly reproducible but has limited sensitivity, which could be supplemented by joining GC–MS analysis. Extracting metabolites from macromolecules requires optimization for trustworthy results. Different extraction methods yield distinct profiles, emphasizing the need for optimization. The results indicated that the optimized extraction procedure successfully identified NMR and GC–MS-based metabolites in MeOH, CHCl_3_, and H_2_O extraction solvents. The findings represented the spectral information related to carbohydrates, organic molecules, and amino acids from the water-soluble metabolites fraction and a series of fatty acid chains, lipids, and sterols from the lipid fraction. Our study underscores the benefit of combining NMR and GC–MS techniques to comprehensively understand microalgae metabolomes, including high and low metabolite concentrations and abundances.

• In this study, we focused on optimizing the extraction procedure and combining NMR and GC–MS techniques to overcome the low NMR sensitivity and the different detected range limits of NMR and GC–MS.
• We explored metabolome diversity in a tropical strain of the small cells’ diatom *Cheatoceros tenuissimus.*

## Graphical abstract

**Figure:**
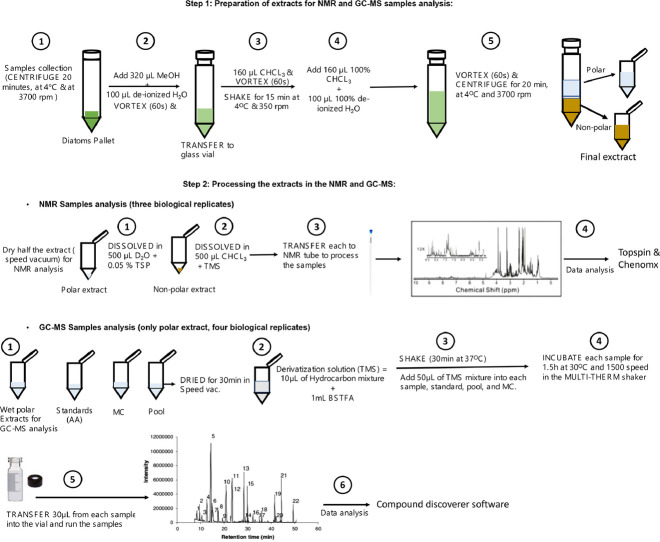

Specifications table

**Table:** 

Subject area:	Environmental Science
More specific subject area:	Marine Science
Name of your method:	Optimizing extraction procedure and combining NMR with GC–MS techniques for comprehensive metabolome analysis of microalgae.
Name and reference of original method:	Name of the method: Extraction strategies for NMR metabolomics from animal tissues. Reference: Lin, C. Y., Wu, H., Tjeerdema, R. S., & Viant, M. R. (2007). Evaluation of metabolite extraction strategies from tissue samples using NMR metabolomics. Metabolomics, 3, 55–67. Link: https://link.springer.com/article/10.1007/s11306-006-0043-1
Resource availability:	800-MHz NMR (Bruker 800 MHz AVANCE) GC–MS (Agilent Technologies) All solvents can be found in ALDRICH and Cambridge Isotope Laboratories. Chenomx NMR Suite 9.0 (Chenomx Inc., Edmonton, Canada) Compound Discoverer software 3.2 (Thermo Fisher Scientific)

## Background

The study aims to optimize reproducible and efficient methods to comprehensively describe the global diversity and profile of key metabolites in the diatom species *Cheatoceros tenuissimus* grown under relatively high tropical temperatures. In addition, the presented extraction procedure consists of a solvent combination of methanol (MeOH), chloroform (CHCl_3_), and de-ionized water (H_2_O), which aims to form two metabolites’ fractions, where the polar in the top and the organic layer in the bottom where many studies struggle to meet these two layers from one extract [1]. Subsequently, we evaluated the efficiency of this solvent mixture for extracting metabolites from *Cheatoceros tenuissimus* with relatively low biomass harvested due to its small cell size (∼ 4–5 µm cell diameter). Also, tropical temperature conditions could modify lipidome [2] and reduce diatom's cell size [3]. *Cheatoceros tenuissimus*, isolated from the Red Sea [4], experiences tropical seawater temperatures in its natural environment, where 26 °C is the average surface seawater temperature [4]. We employed gas chromatography-mass spectrometry (GC–MS) and nuclear magnetic resonance (NMR, 800 MHz) techniques to comprehensively describe the profiles of identified metabolites from water and lipids fractions by improving the extraction procedure.

## Method details

### Culture growth and cell harvesting

*Cheatoceros tenuissimus* was grown under axenic conditions and maintained at 26 °C in f/2 medium + Silicate replicated batch cultures. Illumination was provided under a day/night cycle of 12/12 h with a photon flux density of 400 µmol photons m^−2^ s^−1^. Initial cell concentration was set at 1000 cells mL^−1^, with maximum cell density reaching up to 1.1 × 10^6^ cells mL^−1^ at the stationary phase. Cell abundance was determined through a microscopic examination using a hemacytometer and a LEICA DMI 3000B microscope.

Diatom cells were collected from cultures for extraction around day 8 during the exponential growth phase. At the cell harvesting time, the density of the cell biomass was approximately 3.22 × 10^5^ cells mL^−1^ and a minimum of five million cells in the total collected sample. Four biological replicates of 50 mL each were used for subsequent NMR and GC–MS-based metabolomic analyses. Each 50 mL sample was divided into two fractions (25 mL each) to facilitate supernatant removal and pellet manipulation. Each sample was collected in a 50 mL falcon tube and centrifuged for 20 min at 4 °C and 3700 rpm speed to concentrate the cells and remove seawater. Centrifuge speed was adjusted depending on the sample size (cell biomass) and type (diatom). Tubes were placed directly on ice to maintain a cold environment and slow biological activity. Algal pellets were carefully separated from the media by removing the supernatant. The pellets from the same sample were combined and transferred to small 2 mL Eppendorf cryovials. To ensure the total removal of the media, cell pellets were washed four times by mixing the pellets with 500 µL Milli-Q water in each cryovial, followed by centrifugation for 5 min at 4 °C and 3700 rpm. After removing the liquid, all samples were snap-frozen in liquid nitrogen and stored at −80 °C until extraction.

### Extraction procedure

**Fig. 1:**
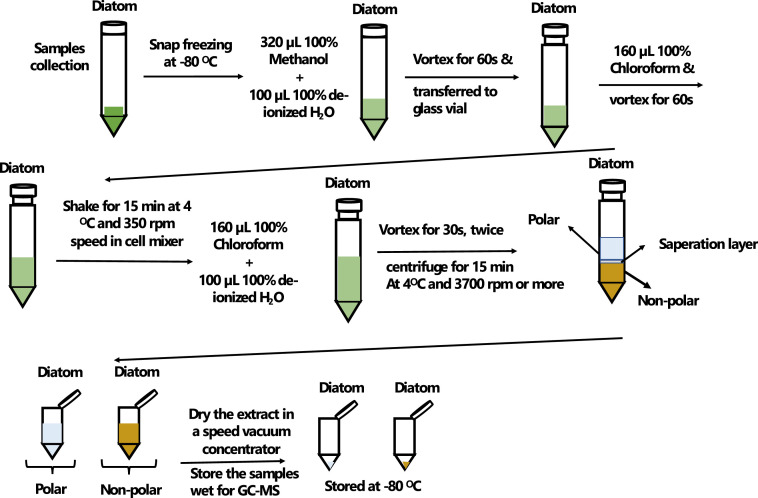
Scheme of the procedure used to extract the polar and non-polar metabolites from diatom species *C. tenuissimus* using extracts solvent mixture of methanol, chloroform, and water.

A detailed extraction protocol has been demonstrated in Fig. 1. To ensure the integrity of the extraction procedure, both samples and solvents were maintained in an ice-cold environment throughout the process. Solvent volumes were adjusted experimentally according to the harvested cell biomass. High-performance liquid chromatography grade (HPLC 99.9% purity) solvents were used exclusively for the extraction.

Initially, 320 µL of 100% MeOH (HPLC grade methanol) and 100 µL de-ionized H_2_O were added to the dried cell pellets, followed by vertexing for 60 s—consistent vortex time maintained throughout the procedure. The mixture was transferred to a small (3 mL) glass vial to mitigate potential interactions between chloroform and plastic. Subsequently,160 µL of CHCL_3_ (HPLC grade chloroform) was added, vortexed for 60 s, then shaken for 15 min at 4 °C and 350 rpm in a cell mixer with shaking speed adjusted according to sample size and type. Primary separation of water and lipid layers was observed at this stage.

An additional 160 µL of chloroform was added after the addition of 100 µL de-ionized H_2_O, with each addition followed by 60 s of vertexing. The mixture was centrifuged for 15 min at 4 °C and 3700 rpm or higher with caution to not tightly close the lid of the falcon tube to prevent tube breakage due to high speed and pressure. Optimal centrifugation time and speed were determined and modified experimentally for the sample size and type used in this study.

Upon completion, the falcon tube was carefully removed using a long tweezer, avoiding sample shaking, and placed in the tube holder inside the ice. Cryovials (2 mL) were prepared, labeled, and opened for sample transfer. Using a glass Pasteur pipette, the polar layer was carefully extracted without disturbing the middle layer. Some top layer was retained to prevent mixing with the middle layer. The lipid layer was then gently collected from the bottom of the vial without disturbing the middle layer. The lipid (∼1 mL) and half of the water extracts (∼500 µL) were dried overnight in a speed vacuum concentrator and stored at −80 °C for later analysis by NMR, with optimum vacuum times and speed used to ensure consistent results across samples. The other half of the water extracts (∼500 µL) were stored wet at −80 °C for later analysis by GC–MS.

### NMR sample preparation and acquisition

We employed a refined protocol, modified from references [5], [6], [7], for preparing samples intended for NMR and GC–MS analysis. For NMR analysis, we first allowed all the dried samples to thaw at room temperature. For polar layer samples, we used 500 µL D_2_O water as a dissolved solvent containing 0.05% Trimethylsilylpropanoic acid (TSP) as a standard. For non-polar samples, we used 500 µL d-chloroform as a dissolved solvent containing tetramethyl-silane (TMS) as an internal reference. Following this. each sample underwent vertexing twice for a minimum of one minute before being transferred into an NMR tube (5 mm in diameter).

The acquisition of the ^1^H NMR spectra for all samples was executed using an 800-MHz NMR (Bruker 800 MHz AVANCE, NEO spectrometer) equipped with TCI cryogenic-probe of ^1^H/^13^C/^15^N (Bruker Bio-Spin, Rheinstetten, Germany). The “zgesgp” pulse program from the Bruker pulse library was used, with over 500 scans conducted to generate each ^1^H NMR spectrum. Subsequently, the obtained spectra underwent modification via Bruker Bio-Spin's Topspin 4.2.0 for phasing and baseline correction. Automatic calibration of each spectrum was performed using the anomeric proton signal of the standards: TSP for polar peaks and TMS for non-polar peaks positioned at 0.00 ppm (chemical shift).

**Fig. 2:**
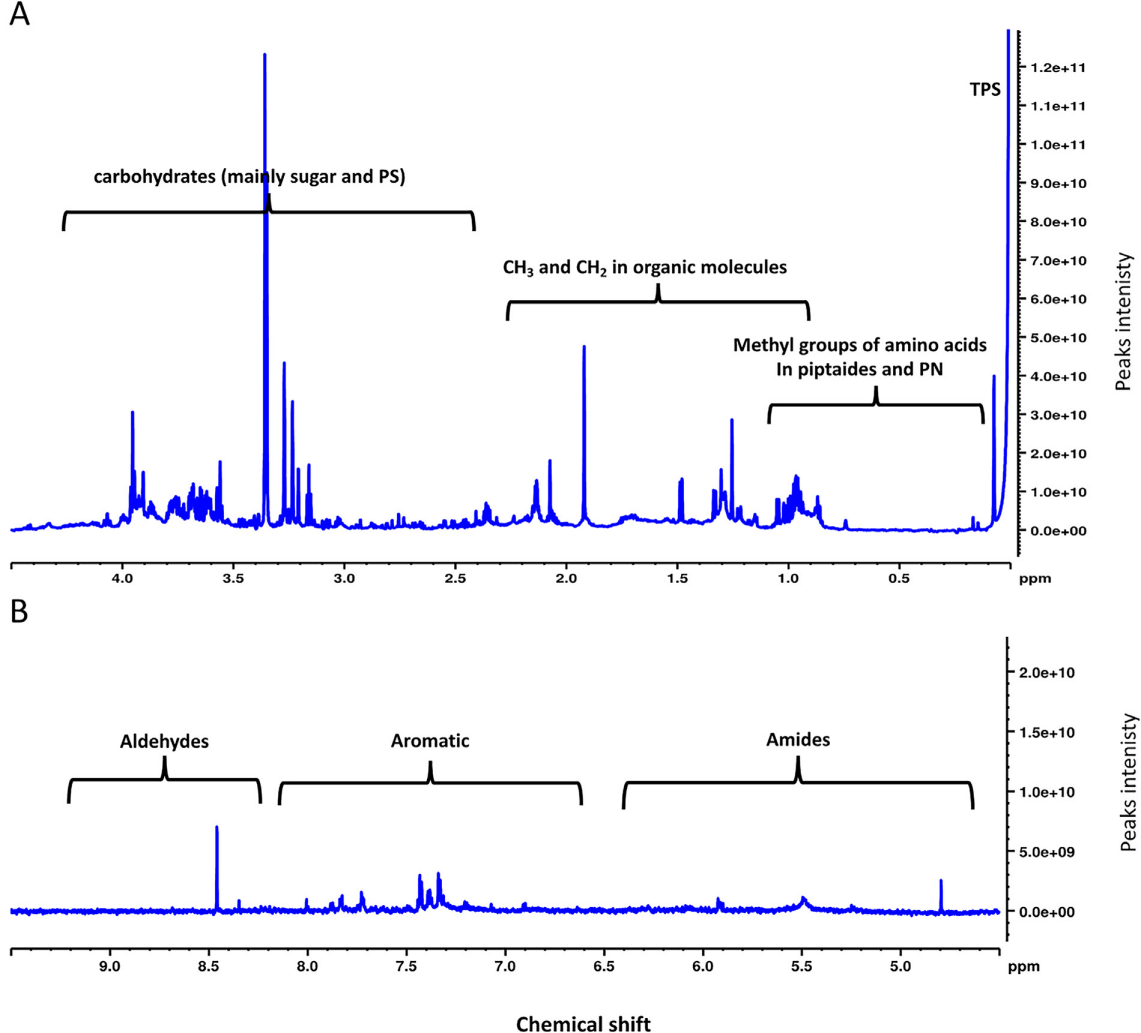
^1^H proton NMR 800-MHz spectral information and assignments of (A) polar assignments of the region between 0.5–4.5 ppm, and (B) the polar assignments of the region between 3.0–9.5 ppm of the diatom *C. tenuissimus* grown under tropical temperature. The X-axis represents the chemical shifts (ppm) of the entire spectra, and the Y-axis represents the intensity values (× 10^9^). PN represents phospholipid nanoparticles, and PS means polysaccharides.

Following calibration, metabolite identification was facilitated using Chenomx NMR Suite 9.0 (Chenomx Inc., Edmonton, Canada), as depicted in Figs. 2**&** 3. To address the multiplicity or overlap of ^1^H NMR signals, peaks corresponding to identified metabolites underwent fitting employing a combination of a local baseline and Voigt functions.

**Fig. 3:**
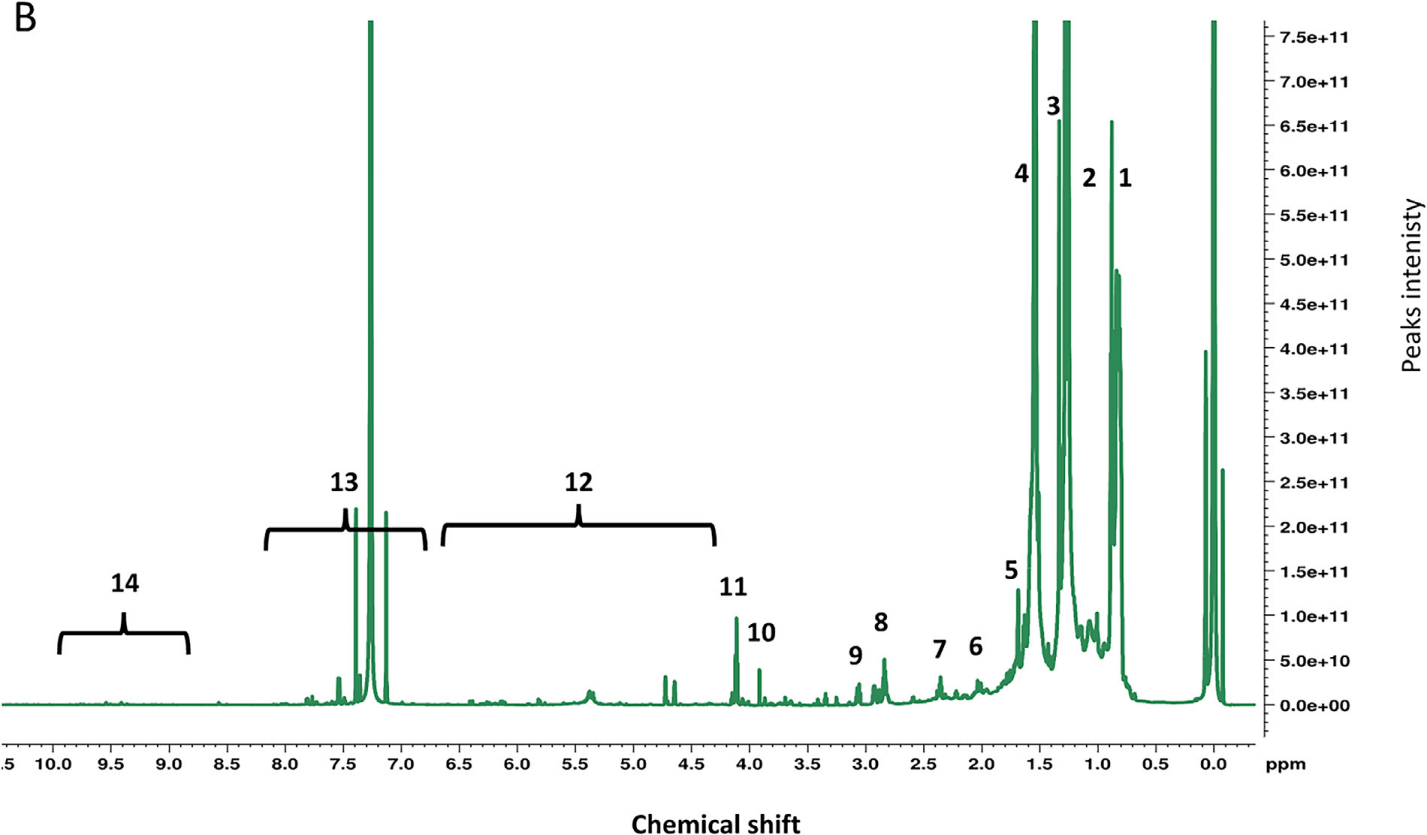
^1^H NMR 800-MHz spectral information and assignments of non-polar layer for the region between 0.5–10.0 ppm for the diatom *C. tenuissimus* grown under tropical temperature. The X-axis represents the chemical shifts of spectra (ppm), and the Y-axis represents the intensity values (× 10^9^). The peaks in the spectra were assigned with the respective functional groups of lipids as ([1]; 0.81 ppm); (CH_3_)n from different types of lipids, ([2]; 0.94 ppm); (CH_3_)k from an adjacent vinyl group of fatty acids, ([3]; 1.22 ppm); (CH_2_)*n-* methyl groups, ([4]; 1.58 ppm); (CH_2_-(CH_2_-)m)*n-* methyl groups, ([5]; 1.62 ppm); (CH_2_-(CH_2_-)o)*n-* methyl groups, ([6]; 2.38 ppm); (OOC—CH_2__—_CH_2_-)n methylene protons adjacent to methylene groups, caroboxylic groups of lipids, ([7]; 2.61 ppm); (—OC—CH_2__—_CH=CH—)n methylene protons adjacent to unsaturated carbons and carboxylic groups in lipids, ([8]; 2.91 ppm); (—N+(CH_3_)_3_)n, ([9]; 3.11 ppm; (-+NCH_2_-), ([10]; 3.39 & 3.62 ppm; (Y-CH_2__—_CH_2_-X) and (Y-CH_2__—_CH_2_-X) found in sterols, ([11]; 4.13 ppm); (—CHOH—)n, ([12]; 5.80–6.60 ppm); Al(CH=CH—)n, ([13]; 7.15–7.82 ppm; Aromatic (Ar)(—CH=CH—)n finds in aromatic alkaloids, and ([14]; 9.97 ppm); (HOOC-)n found in fatty acids. Aliphatic unsaturated double bonds are found in the unsaturated fatty acids, phospholipids (PL), phosphatidylcholine (PC), triglycerides (TG), and alkyl hydroxy groups found in cholesterol. Methyl groups found at different numbers of TG, Lipids, Cholesterol, and the N+(CH_3_)_3_ groups found in resonances of sphingomyelin (SM) and phosphatidylcholine [same as above].

### GC–MS sample preparation and acquisition

For GC–MS acquisition,100 µL aliquots were transferred from each wet water layer sample into individual cryovials. Additionally, a range of different amino acid mixture concentrations (1 µL, 2.5 µL, 5 µL, 10 µL, 25 µL, 50 µL) were prepared as internal standards. Three replicates of empty cryovial containing no samples served as method controls (MC). Pool samples (4 replicates) were prepared by combining aliquots from all replicates (100 µL each) and served as quality controls. Subsequently, all prepared samples were dried for 30 min in a speed vacuum.

GC–MS faces a limitation wherein the analyst metabolites must be volatile and thermally stable [8]. This poses a challenge as many metabolites, especially polar compounds, do not meet these criteria. To overcome this, derivatization agents, such as trimethylsilyl (TMS) groups, are commonly employed to enhance volatility and thermal stability and reduce polarity [8]. A derivatization solution was prepared by mixing 10 µL of hydrocarbon mixture (C7-C40) with 1 mL of BSTFA [N, O-Bis(trimethylsilyl)trifluoroacetamide]. The derivatized agent was then shaken for 30 min at 37 °C and 1500 rpm in a multi-thermal shaker (Benchtop, Benchmark Scientific Inc.).

Subsequently, 50 µL from the derivatization agent mixture was added to each dried sample, pool, standards, and MC. The cryovials were then incubated for 1.5 h at 30 °C and 1500 rpm speed in the multi-thermal shaker. From each sample, 30 µL aliquots were transferred into gas chromatography (GC) vial deactivated inserts and injected into the GC–MS analyzer.

GC–MS spectra processing and all statistical analysis were conducted using Compound Discoverer software. Processing steps included the imputation method performed with the Random Forest algorithm (MissForest algorithm, [9]). Quality control correction was carried out as per established protocols [10]. Each batch analysis allowed a maximum of 15 files, with samples analyzed in a single batch. Samples were normalized to the maximum peak area mean of all samples to ensure consistency in data analysis.

## Results

### NMR-based metabolites identification in extraction solvents

**Fig. 4:**
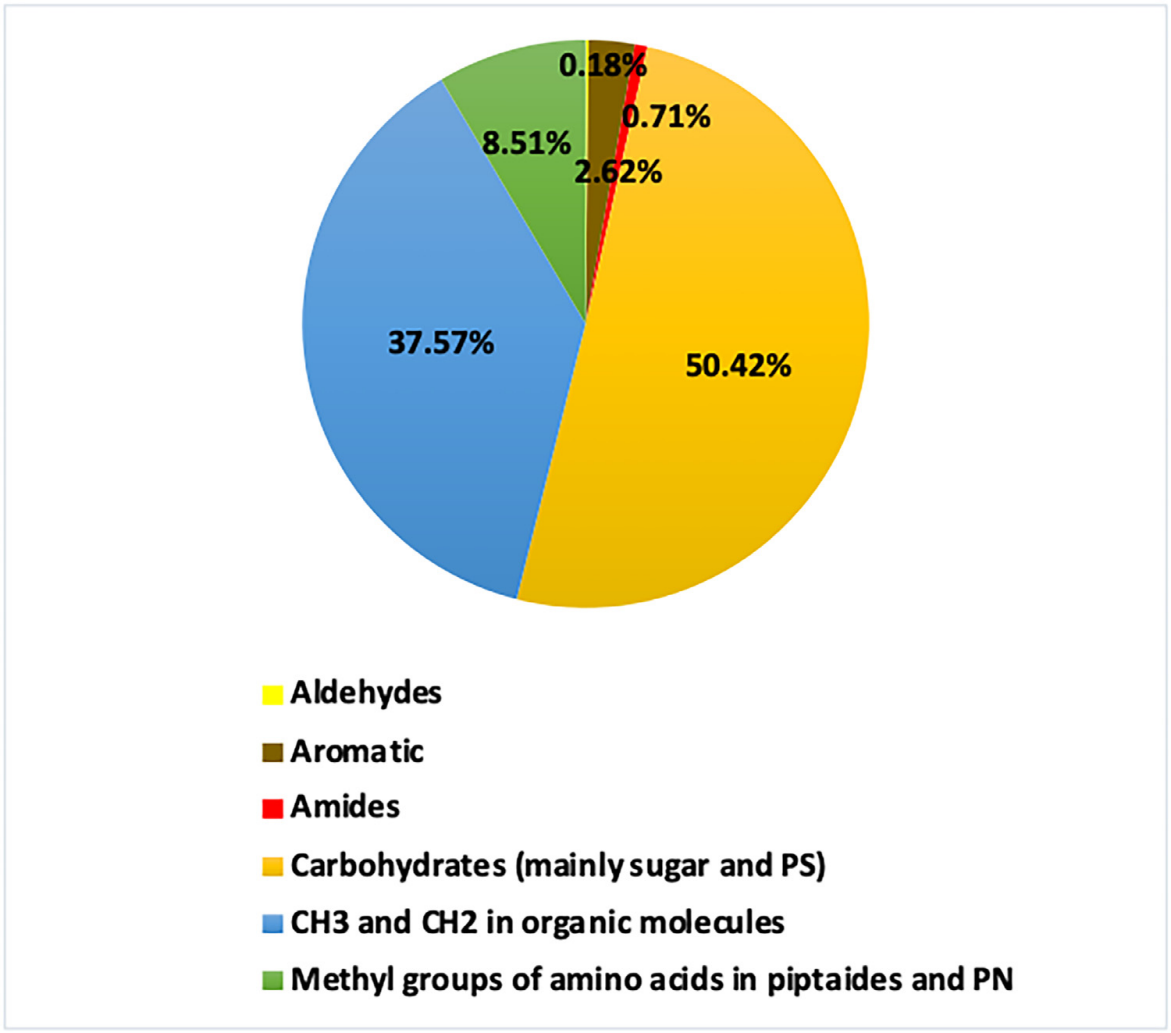
Pie chart shows the percentage contribution of each integrated region from *C. tenuissimus* spectra grown under tropical temperature.

The spectral information and assigned regions of *C. tenuissimus’* polar layer using the proposed extract procedure have been successfully obtained (Fig. 2). Peaks in marine algae [11] are typically assigned as methyl groups found in amino acids within peptides and phospholipid nanoparticles (PN) in the range of 0.8 to 1.1 chemical shift (ppm), while peaks falling between 0.8 and 2.3 ppm are commonly associated with CH_3_ and CH_2_ groups in organic molecules. In the 2.2 to 5.2 ppm range, we generally find peaks related to carbohydrates, primarily sugars and polysaccharides (PS). Amide peaks appear between 5.5 and 6.6 ppm, while aromatic peaks are typically between 7.0 and 8.4 ppm. Peaks further down the field at 8.0 to 10.0 ppm are indicative of aldehydes. In general, carbohydrates contributed to most of the integrated regions among other classes, with 50.42%, followed by organic molecules and amino acids (Fig. 4). Different classes of amides, aromatic and aldehyde integrated regions contributed the minimum (Fig. 4). Using Chenomx software, we found that the lower concentration detected by NMR metabolomics-based analysis was 0.0001 mM, belonging to choline amino acid and its family, while the highest concertation reported was 0.02 mM belong to acetate organic molecule. We further assigned the non-polar spectra of the lipids extracts from the regions between ∼0.5–10.0 following assignments outlined by several studies [12], [13], [14], [15] (Fig. 3). Mostly we found different types of lipids from an adjacent methyl and vinyl group of fatty acids, sterols, aromatic finds in aromatic alkaloids1, and aliphatic unsaturated double bonds found in the unsaturated fatty acids, phospholipids (PL), phosphatidylcholine (PC), triglycerides (TG), alkyl hydroxy groups found in cholesterol, and the N+(CH_3_)_3_ groups found in resonances of sphingomyelin (SM), and phosphatidylcholine [12], [13], [14], [15]. In general, lipids had higher concentrations than water-soluble metabolites, ranging between 0.01 mM to a maximum of 0.97 mM, belonging to several methyl groups found at different numbers of triglycerides, lipids, and cholesterol. Our method identified a broad spectrum of fatty acid and sterol metabolites in the non-polar layer, accounting for lipid metabolites in the organic layer, which is sometimes hard to obtain sufficiently from the same extract.

### GC–MS-based metabolites identification in extraction solvents

We combined the state-of-the-art ^1^H proton NMR spectroscopy and GC–MS techniques to evaluate the metabolome diversity of tropical *C. tenuissimus* comprehensively. GC–MS metabolomic-based analysis allowed a wide range of metabolites to be identified and presented at much lower concentrations than NMR. Through rigorous scrutiny of the metabolic data and utilizing the metabolic databases available within the Compound Discoverer software framework, we have successfully delineated a discerning compilation of 258 identified and unknown metabolites (Table 1). Compared to a prior investigation on the diatom *C. tenuissimus*, where they identified 43 metabolites from the polar layer [1], our approach revealed a significantly higher number of metabolites, ranging from 126 identified metabolites to 128 unknown metabolites from the polar fraction (Table 1). Moreover, the GC–MS data analysis revealed that using the proposed method, we identified metabolites as low abundance as 10^3^, which mainly belong to amino acids. In contrast, the highest abundant metabolites detected reached up to 10^10^, mostly organic molecules. On the other hand, NMR metabolite's lowest abundance was 10^9^, belonging to the sugar and carbohydrates classes, while the maximum metabolite abundance reached up to>10^11^, covering a wider range of organic molecules present at higher concentrations. This indicates that combining NMR and GC–MS techniques is better [10] for a broad and comprehensive understanding of the metabolome diversity of microalgae grown under specific conditions.

**Table 1:** List of water-soluble primary metabolites observed by GC–MS analysis from the Compound Discoverer Library describing names, retention time (RT), and their peaks area abundances of metabolites obtained for the tropical strain of *C. tenuissimus* growing at 26 °C.

Name	RT [min]	Area abundance
Ethanolamine, 3TMS	6.015	1.12E+07
1-Hexyl-2-nitrocyclohexane	6.031	3.69E+07
Nonane	6.369	8.52E+08
Silane, dimethyl(dimethyl(3-phenylpro-2-enyloxy)silyloxy)(3-phenylpro-2-enyloxy)-	6.443	6.00E+06
L-Valine, 2TMS	6.535	6.98E+04
5-(2-Aminoethyl)thiophene-2-sulfonamide, N,N,N',N'-tetrakis(trimethylsilyl)-	6.786	4.60E+08
Fladrafinil	7.059	2.09E+08
Benzoic acid, 1TMS	7.090	1.47E+07
Leucine, 2TMS	7.413	7.92E+06
Silanol, trimethyl-, phosphate (3:1)	7.438	1.63E+10
Silanol, trimethyl-, phosphate (3:1)	7.464	7.29E+09
Silanol, trimethyl-, phosphate (3:1)	7.563	2.18E+09
Silanol, trimethyl-, phosphate (3:1)	7.648	8.49E+07
L-Allo-isoleucine, 2TMS	7.823	7.00E+05
Proline, 2TMS	7.872	1.40E+05
Oxalic acid, allyl decyl ester	7.974	8.77E+08
Glycine, 3TMS	7.982	9.22E+06
Pyrrolo[1,2-a]quinoline-1-ethanol, dodecahydro-6-(2,4-pentadienyl)-, [1R-[1α,3aβ,5aα,6α(Z),9aα]]-	8.019	8.49E+06
Peak@8.051	8.050	3.32E+08
2,2,3,3,4,4,4-Heptafluoro-N-[[4-[(2,2,3,3,4,4,4-heptafluorobutanoylamino)methyl]phenyl]methyl]butanamide	8.285	6.27E+06
L-Serine, 3TMS	8.774	4.08E+06
L-Serine, 3TMS	8.913	2.87E+06
L-Threonine, 3TMS	9.306	8.02E+05
DL-Threonine, 3TMS	9.315	2.58E+05
L-Aspartic acid, N-trimethylsilyl-, 4-methyl ester, 1-trimethylsilyl ester (isomer 1)	9.522	9.63E+04
1-Nonene, 4,6,8-trimethyl-	9.584	8.53E+08
L-Aspartic acid, N-trimethylsilyl-, 4-methyl ester, 1-trimethylsilyl ester (isomer 1)	9.680	5.90E+05
Terephthalic acid, di(2-methylphenyl) ester	9.754	2.77E+07
L-Aspartic acid, N-trimethylsilyl-, 1-methyl ester, 4-trimethylsilyl ester (isomer 2)	9.794	2.68E+04
L-Aspartic acid, N-trimethylsilyl-, 1-methyl ester, 4-trimethylsilyl ester (isomer 2)	9.974	1.69E+04
3-Amino-2-piperidone, 2TMS derivative	10.210	6.26E+04
Peak@10.358	10.362	2.59E+06
3-Amino-2-piperidone, 2TMS derivative	10.410	5.01E+04
Pyruvic acid, 2TMS	10.412	1.34E+06
tert-Butyldimethylsilyl 2,3-dimethylbenzoate	10.815	7.24E+06
tert-Butyldimethylsilyl 2,3-dimethylbenzoate	10.958	4.68E+08
Methyl l-alaninate, 2TMS derivative	11.067	7.78E+05
Pentane, 2,2,3,4-tetramethyl-	11.144	8.80E+08
L-Methionine, 2TMS	11.156	5.36E+05
Pyroglutamic acid, 2TMS	11.165	1.12E+06
Butylated Hydroxytoluene	11.191	2.21E+08
Propylene glycol, 2TMS	11.195	6.56E+07
DL-Aspartic acid, 3TMS	11.207	5.88E+07
Tromethamine, 4TMS derivative	11.276	6.25E+07
L-Methionine, 2TMS	11.356	7.09E+06
Pyroglutamic acid, 2TMS	11.381	2.75E+06
L-Aspartic acid, 3TMS derivative	11.404	1.02E+06
L-Aspartic acid, 3TMS	11.406	2.40E+06
Furethidine	11.612	1.43E+04
3-Hydroxy-N-(1‑hydroxy-4-methylpentan-2-yl)−5-oxo-6-phenylhexanamide, 3TMS	11.665	1.01E+07
2H-1,4-Oxazinimine, 3,4,5,6-tetrahydro-3,3,4,5,5-pentamethyl-N-(2,4,6-trinitrophenyl)-	11.720	2.78E+06
Cysteine, 3TMS	11.890	6.47E+04
1-Octanol, 2‑butyl‑	12.617	1.14E+09
L-Glutamic acid, 3TMS	12.672	7.84E+03
L-Phenylalanine, 2TMS	12.674	1.14E+04
L-Phenylalanine, 2TMS derivative	12.732	5.23E+04
L-Ornithine, 3TMS derivative	12.802	4.99E+04
L-Glutamic acid, 3TMS	12.869	1.72E+06
L-Phenylalanine, 2TMS	12.904	5.51E+06
2,4,6-Tris(1,1-dimethylethyl)−4-methylcyclohexa-2,5‑dien-1-one	13.311	1.21E+06
2-naphthalenecarboxamide, N-hexadecyl-1‑hydroxy-	13.39	2.38E+06
Phenol, 2,6-bis(1,1-dimethylethyl)−4-methyl-, methylcarbamate	13.51	1.45E+06
(Z)−5-Methoxy-3,5-dimethyl-2-hexenyltrimethylsilane	13.553	6.23E+06
1,5-Octadiene, 7-methyl-3-(1-methylethyl)-	13.912	2.87E+07
Cyclohexane, 2,4-diisopropyl-1,1-dimethyl-	13.920	2.19E+08
Oxalic acid, allyl hexadecyl ester	14.019	1.24E+09
L-Lysine, 3TMS derivative	14.071	1.38E+04
Propane, 1,3-bis(dimenthyphosphino)-	14.12	2.73E+05
N-(2-Nitrophenyl)ethylenediamine, 2TMS derivative	14.309	5.09E+07
8-Methoxy-2-(p-methoxyphenyl)−1,2,4,5-tetrahydro-1-benzazocine-3,6-dione	15.080	3.52E+04
Oxalic acid, allyl hexadecyl ester	15.355	1.20E+09
L-Ornithine, 4TMS	15.500	2.30E+04
Citric acid, 4TMS	15.550	7.69E+05
L-Arginine, 3TMS	15.629	8.62E+03
N-α-Acetyl-l-Lysine, 3TMS derivative	15.880	6.91E+03
Timonacic, 2TMS derivative	15.994	5.67E+03
Silane, tetramethyl-	16.045	3.54E+06
L-Phenylalanine, 3TMS derivative	16.188	1.70E+07
Tyrosine, 2TMS derivative	16.378	2.29E+04
3-Heptene, 2,2,4,6,6-pentamethyl-	16.396	1.30E+07
(Z)−5-Methoxy-3,5-dimethyl-2-hexenyltrimethylsilane	16.418	1.28E+05
(Z)−5-Methoxy-3,5-dimethyl-2-hexenyltrimethylsilane	16.457	4.85E+05
3-Heptene, 2,2,4,6,6-pentamethyl-	16.511	1.11E+06
L-Histidine, 3TMS	16.559	2.41E+05
Oxalic acid, allyl hexadecyl ester	16.587	2.61E+08
Decane, 2,3,5,8-tetramethyl-	16.634	8.34E+08
L-Lysine, 4TMS	16.865	1.95E+05
Tyrosine, 3TMS	16.886	3.23E+05
Tyrosine, 3TMS	17.055	2.21E+05
Arabinose isomer 1, 4TMS	17.340	7.05E+05
Decane, 2,3,5,8-tetramethyl-	17.860	7.78E+08
Heptadecane, 2,6-dimethyl-	17.882	7.39E+08
Propane, 2‑bromo-2-methyl-	18.086	1.76E+06
D-Chiro-Inositol, 6TMS	18.111	1.20E+04
Palmitic acid, 1TMS	18.385	1.00E+08
Oxalic acid, allyl octadecyl ester	19.019	1.33E+09
1-Nonene, 4,6,8-trimethyl-	19.041	2.29E+08
Oxalic acid, allyl octadecyl ester	20.068	1.33E+09
L-Tyrosine, N,N-di-acetyl, O,O-bis-TMS	20.591	1.09E+06
L-Cystine, 4TMS	20.654	8.41E+03
L-Cystine, 4TMS	20.841	1.81E+06
Heptadecane, 2,6-dimethyl-	20.940	1.26E+09
Melibiose isomer 1, 8TMS	21.019	4.31E+07
Butane-1,3-diol, 1-methylene-3-methyl-, bis(trimethylsilyl)ether	21.145	8.04E+05
Oxalic acid, allyl octadecyl ester	21.676	1.26E+09
Heptadecane, 2,6-dimethyl-	22.323	1.26E+09
Octane, 2-iodo-	22.341	1.20E+07
1-(2-Methoxyethoxy)−2-methyl-2-propanol, TMS derivative	22.465	7.38E+05
Oxalic acid, allyl octadecyl ester	22.905	1.30E+09
Sucrose, 8TMS	23.054	1.33E+07
Maltose isomer 1, 1MOX, 8TMS	23.325	1.10E+06
Isomaltose isomer 2, 1MOX, 8TMS	23.416	1.32E+05
Carbonic acid, eicosyl vinyl ester	23.451	1.29E+09
Decane, 5-ethyl-5-methyl-	23.453	1.03E+07
Pinacol, 2TMS derivative	23.558	6.20E+05
Isomaltose isomer 2, 1MOX, 8TMS	23.568	3.34E+05
Maltose isomer 2, 1MOX, 8TMS	23.718	1.08E+06
1,2-Ethenediol, 2TMS derivative	23.875	1.13E+04
butanamide, N-(3,5-difluoro-2‑hydroxy-4-methylphenyl)−4-(3-pentadecylphenoxy)-	23.905	2.83E+05
Oxalic acid, allyl octadecyl ester	23.937	1.30E+09
Heptadecane, 2,6-dimethyl-	24.405	1.25E+09
Piperazine, 1-ethyl-4-(4-piperidyl)-	24.414	9.28E+06
Silane, [(dimethylsilyl)methyl]trimethyl-	24.496	4.58E+05
Carbonic acid, eicosyl vinyl ester	24.858	1.30E+09
Carbonic acid, eicosyl vinyl ester	25.281	7.14E+08
Melibiose isomer 1, 8TMS	25.327	6.37E+06
2-Methyltetracosane	25.680	1.21E+09
2-Methyltetracosane	26.078	1.12E+09
2-Methyltetracosane	26.483	1.02E+09
Cyclononasiloxane, octadecamethyl-	26.687	3.47E+05
2-Methyltetracosane	26.926	8.84E+08
Carbonic acid, eicosyl vinyl ester	27.423	7.69E+08
2-Methyltetracosane	27.989	5.59E+08

## Additional information

Metabolomics is a recognized omics science focusing on the characterization of originating low-molecular-weight metabolites present in different biological systems [16,17]. This approach plays a crucial role in evaluating the physiological status of biological systems, including algae. The primary producers in the oceanic ecosystem are photosynthetic phytoplankton, composed of algae [18]. Diatoms are a relevant component of algal photosynthetic phytoplankton, which are present in aquatic habitats globally. In marine ecosystems, diatoms contribute about 35–75% of primary productivity [19]. The metabolomic approach has been widely used to assess changes in the metabolite profiles of different diatom species and growth conditions and their response to diverse stress conditions [20,21]. Recent studies involving both metabolomics and transcriptomics have been conducted on the diatom species *Phaeodactylum tricornutum*, revealing that elevated temperatures primarily result in increased concentrations of 2-oxoglutarate, a central molecule in nitrogen and carbon metabolism [22]. This led to heightened fatty acid metabolism, glutamine and glutamate production, urea cycle activity, and the tricarboxylic acid cycle (TCA) [22]. Another metabolomic method was used to find the key metabolites that differentiate the two-growth exponential and stationary phases of the diatom species *Cheatoceros tenuissimus* [1]. Another metabolomics offers significant potential for unravelling changes in metabolism within diatom cells, shedding light on their physiological condition, particularly in response to stressors like temperature. Metabolite extraction is an essential step for sample preparation for comprehensive metabolomics studies. Different extraction methods led to different metabolomics profiles, where effective extraction procedures for some types of samples would not be optimum for other types of samples [5]. Thus, it is crucial to optimize the metabolites extraction method that allows researchers to obtain the highest number of extracted metabolites.

Over the past decades, nuclear magnetic resonance (NMR) has consistently ranked among the top three analytical methods in microalgae metabolomics, alongside gas chromatography-mass spectrometry (GC–MS) [8,23,24] and liquid chromatography-mass spectrometry (LC-MS) [24]. While many studies on diatom metabolites profiling have primarily utilized GC–MS [8,25,26], NMR offers distinct advantages, particularly as a non-destructive, nonbiased, and highly reproducible method [5,27]. This makes NMR valuable for detecting highly concentrated metabolites [25,26]. Peak overlapping and low sensitivities are the main limitations of NMR approaches in metabolomics studies. GC–MS and LC-MS are more sensitive methods that often exhibit a detection range 10–100 times higher than that of NMR, but these two methods can't detect very high metabolite concentrations [28]. The efficiency of NMR in identifying substances is compromised when compounds are present at low concentrations [25,26]. Typically, NMR detects metabolites below several µM, whereas GC–MS can detect concentrations as low as 10 nM [28]. Thus, combining both NMR and MS analytical platforms is highly recommended to maximize the number of detected and identified metabolites.

Moreover, utilizing an NMR metabolomics study requires relatively high biomass, necessitating additional sample size, which could be challenging in specific samples such as algae [28]. Beyond the challenge of low algal biomass, the choice of extraction solvents poses another hurdle in microalgae metabolomics studies, as the effectiveness of metabolite detection depends on the solvent constituents and ratios in the employed extraction method [5,29,30]. Previous research has shown that a solvent mixture of methanol, ethanol, and chloroform in a 1:3:1 ratio effectively detects metabolites in different growth phases of the planktonic marine diatom *Skeletonema marinoi* using the GC–MS technique [29]. However, another commonly used solvent mixture in metabolomics studies from biological tissues, comprising methanol (MeOH), water (H_2_O), and chloroform (CHCl_3_) in a 5:2:2 ratio, used to investigate metabolites profiling using GC and LC-MS techniques [31,32]. In addition, a study has concluded that the preferred extraction method to analyse metabolomics from different fish tissues using NMR techniques as methanol, chloroform, and water mixture, with a volume ratio of 2:2:1.8 as it efficiently yields both hydrophilic and hydrophobic metabolites with high reproducibility [5].

This study described an optimized extraction method to overcome common issues in metabolomics studies on microalgae, including low biomass, low metabolite concentrations, and sufficient amounts of both polar and non-polar fractions, together with different detection ranges of metabolites by NMR or GC–MS [5,26,27]. Despite these challenges, our study successfully used an optimized extraction procedure combining both NMR and GC–MS techniques to comprehensively determine the biodiversity of the key primary metabolites from both polar and non-polar fractions in the diatom species *C. tenuissimus* grown under tropical Red Sea seawater temperature. However, the current study has a few limitations. We needed to increase the scans of the NMR spectra up to 500 to distinguish the assigned peaks from the noise clearly and increase the detection sensitivity. Therefore, we recommend using at least double biomass when using such a method and type of sample. Another limitation is the low concentration and almost disappearance of some classes of metabolites, which could be related to the time of cells’ harvesting as some metabolites could be higher in the stationary phase compared to the exponential phase [1]. Therefore, we recommend exploring the two different growth phases when investigating the diversity of the key metabolites on microalgae grown under specific conditions. We further shell emphasized that to ensure the suitability of this method, we experimentally optimized the extraction procedure to extract the metabolites from the diatom species *C. tenuissimus*. Therefore, we recommend experimentally optimizing important steps such as solvent volumes, centrifugation time, and speed if a different sample size (lower or higher biomass) or type (rather than diatom) is used for this method. The method presented here involves a few steps, which may introduce personal inconsistency. Therefore, researchers should be trained to follow the method strictly with all samples, including the time of harvesting, following sample preparation, and instrumental conditions.

## Ethics statements

The authors confirm that this research doesn't involve any human or animal subjects and doesn't involve any data collected from social media platforms.

## CRediT authorship contribution statement

**Afrah Alothman:** Data curation, Formal analysis, Funding acquisition, Investigation, Methodology, Visualization, Writing – original draft, Writing – review & editing. **Abdul-Hamid Emwas:** Data curation, Resources, Software, Writing – review & editing, Methodology, Funding acquisition. **Upendra Singh:** Data curation, Formal analysis, Funding acquisition, Methodology, Writing – review & editing. **Mariusz Jaremko:** Data curation, Resources, Software, Writing – review & editing, Methodology, Funding acquisition, Supervision, Validation, Project administration. **Susana Agusti:** Conceptualization, Project administration, Supervision, Validation, Writing – review & editing, Data curation, Funding acquisition.

## Declaration of competing interest

The authors declare that they have no known competing financial interests or personal relationships that could have appeared to influence the work reported in this paper.

## Data Availability

Data will be made available on request.
